# MicroRNA-21 Exhibits Antiangiogenic Function by Targeting RhoB Expression in Endothelial Cells

**DOI:** 10.1371/journal.pone.0016979

**Published:** 2011-02-10

**Authors:** Céline Sabatel, Ludovic Malvaux, Nicolas Bovy, Christophe Deroanne, Vincent Lambert, Maria-Luz Alvarez Gonzalez, Alain Colige, Jean-Marie Rakic, Agnès Noël, Joseph A. Martial, Ingrid Struman

**Affiliations:** 1 Unit of Molecular Biology and Genetic Engineering, GIGA-Research, University of Liège, Sart Tilman, Liège, Belgium; 2 Laboratory of Connective Tissues, GIGA-Research, University of Liège, Sart Tilman, Liège, Belgium; 3 Laboratory of Tumor and Development Biology, GIGA-Research, University of Liège, Sart Tilman, Liège, Belgium; 4 Department of Ophthalmology, University Hospital, Sart Tilman, Liège, Belgium; Istituto Dermopatico dell'Immacolata, Italy

## Abstract

**Background:**

MicroRNAs (miRNAs) are endogenously expressed small non-coding RNAs that regulate gene expression at post-transcriptional level. The recent discovery of the involvement of these RNAs in the control of angiogenesis renders them very attractive in the development of new approaches for restoring the angiogenic balance. Whereas miRNA-21 has been demonstrated to be highly expressed in endothelial cells, the potential function of this miRNA in angiogenesis has never been investigated.

**Methodology/Principal Findings:**

We first observed in endothelial cells a negative regulation of miR-21 expression by serum and bFGF, two pro-angiogenic factors. Then using *in vitro* angiogenic assays, we observed that miR-21 acts as a negative modulator of angiogenesis. miR-21 overexpression reduced endothelial cell proliferation, migration and the ability of these cells to form tubes whereas miR-21 inhibition using a LNA-anti-miR led to opposite effects. Expression of miR-21 in endothelial cells also led to a reduction in the organization of actin into stress fibers, which may explain the decrease in cell migration. Further mechanistic studies showed that miR-21 targets RhoB, as revealed by a decrease in RhoB expression and activity in miR-21 overexpressing cells. RhoB silencing impairs endothelial cell migration and tubulogenesis, thus providing a possible mechanism for miR-21 to inhibit angiogenesis. Finally, the therapeutic potential of miR-21 as an angiogenesis inhibitor was demonstrated *in vivo* in a mouse model of choroidal neovascularization.

**Conclusions/Significance:**

Our results identify miR-21 as a new angiogenesis inhibitor and suggest that inhibition of cell migration and tubulogenesis is mediated through repression of RhoB.

## Introduction

Over the past few decades, microRNAs (miRNAs) have emerged as a prominent class of gene regulators. miRNAs are endogenous small single-strand non-coding RNAs of about 22 nucleotides [1]. Together with a protein complex known as RNA-induced silencing complex (RISC), miRNA functions as a guide molecule in post-transcriptional gene silencing by partially complementing to the 3′ untranslated region (UTR) of target mRNA. Base-pairing between miRNA and mRNA leads to translational repression or mRNA degradation [2]. To date, about 1000 miRNAs have been identified in humans. Each miRNA can regulate up to hundreds of targets and miRNAs are predicted to regulate a third of protein coding genes [3]. By regulating numerous mRNAs, miRNAs play a key role in a wide range of physiological and pathological processes [4], [5].

Accumulating evidence indicates that aberrant miRNA expression profiles are observed in a variety of cancers. Among the many miRNAs already identified as regulators of neoplastic transformation, invasion and metastasis, miR-21 has emerged as a key miRNA dysregulated in many cancers [6]. miR-21 has been reported to be highly expressed in breast [7], lung [8], pancreas [9], prostate [10], stomach [10] and brain [11] cancers. An increased expression of miR-21 in tumor cells is associated with a higher proliferation and invasion capacity of these cells [12]. Moreover, elevated miR-21 expression in cancer has been shown to be generally associated with poor prognosis [13].

Despite the numerous studies performed regarding the role of miR-21 in tumor cells, its functions in endothelial cells remain to be defined. Indeed, *de novo* angiogenesis is a critical factor in cancer progression. Several specific miRNAs control blood vessel formation, such as miR-126 [14]. This endothelial specific miRNA promotes blood vessel development by targeting negative regulators of MAP kinase and PI3K signaling pathways [15], [16]. miRNAs, regulating key angiogenic processes, are defined as angiomiRs and to date, only a few angiomiRs have been described [17]. Of these angiomiRs, some negatively regulate angiogenesis. For example, miR-221 and miR-222 have been shown to block angiogenesis due to their regulatory effect on the stem cell factor receptor c-Kit [18]. In addition, the miR-17-92 cluster has also been identified as a negative regulator of angiogenesis. In this cluster, miR-92a inhibits angiogenesis both *in vitro* and *in vivo* [19]. More recently, miR-17/20 was shown to exert cell intrinsic antiangiogenic activity [20].

Aberrant angiogenesis leads to a variety of pathologies such as cancer, ischemia, psoriasis and age-related macular degeneration. The angiogenesis status is defined by a balance between pro- and antiangiogenic molecules. This homeostasis is perfectly controlled by a tight regulation of the level of angiogenesis inducers and inhibitors, and any dysregulation is often associated with pathological settings [21]. Further understanding of the orchestration of this balance could help in the development of strategies to harness the dynamics of blood vessels in human health and diseases. Identification of miRNAs controlling angiogenesis is thus a very interesting approach based on their broad-spectrum effects. Due to its central role in cancer progression and to the close association between tumor progression and angiogenesis, we suggest that miR-21 may be involved in angiogenesis. Moreover, analysis of miRNAs expressed in endothelial cells has revealed that miR-21 is one of the most highly expressed miRNAs in these cells [22], [23]. In addition, among the different genes reported to be targets of miR-21, some have been identified as angiogenesis modulators. The regulator of MAPK activation Sprouty1, whose antiangiogenic potential has already been demonstrated [24], is targeted by miR-21 in cardiac fibroblasts [25]. Moreover, a recent study performed in hepatocellular carcinoma cell lines demonstrated a direct interaction between miR-21 and the 3′UTR of RhoB, a gene that may be involved in angiogenesis regulation [26].

In the current study, we first performed *in vitro* experiments to test the involvement of miR-21 in angiogenesis. From our data, miR-21 emerges as a new negative regulator of angiogenesis. Our results also provide a possible mechanism for miR-21 to inhibit angiogenesis via RhoB targeting. Moreover, our *in vivo* study demonstrates the therapeutic efficacy of miR-21 overexpression in reducing angiogenesis in a model of pathological choroidal neovascularization.

## Results

### Regulation of miR-21 expression by growth factors in endothelial cells

**Figure 1:**
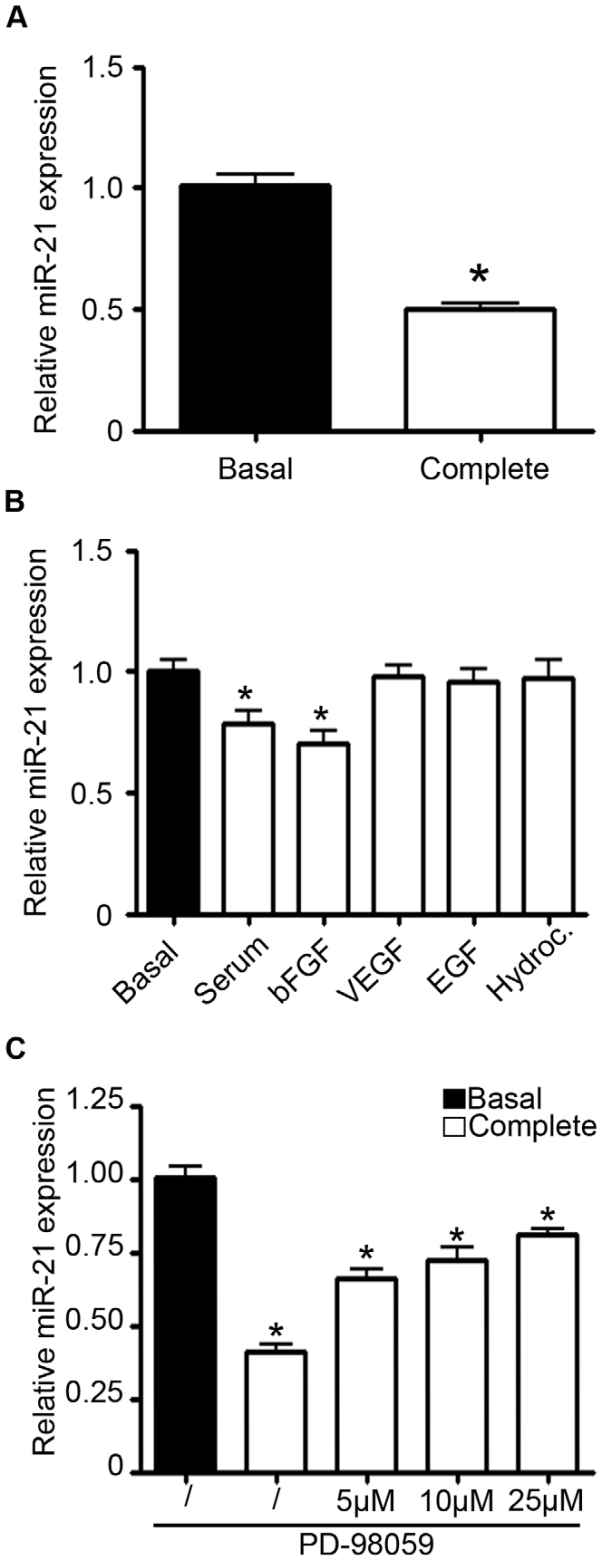
Regulation of miR-21 expression by growth factors in endothelial cells. **A–C**. HUVECs were cultured in basal medium (EBM) only, basal medium supplemented with a cocktail of angiogenic factors (EGM) or different amounts of angiogenic factors (serum 5%, bFGF 10 ng/ml, VEGF 50 ng/ml, EGF 20 ng/ml and hydrocortisone 1 ng/ml). After 72 hours, miR-21 expression was quantified by qRT-PCR. **C**. HUVECs were cultured in EBM or in EGM supplemented with increasing concentrations of PD-98059. miR-21 expression was quantified by qRT-PCR after 72 hours. **A–C**. The data were normalized to RNU-44 and converted using the formula 2^−ΔΔCt^ (relative expression). Data are means with the SD. *p<0.05 versus corresponding control; (n = 3).

Several studies have previously demonstrated a high expression level of miR-21 in endothelial cells [22]. In order to determine a potential role for miR-21 in angiogenesis, we analyzed the impact of growth factor treatment on miR-21 expression in human umbilical vein endothelial cells (HUVECs). miR-21 abundance was first analyzed in HUVECs cultured in endothelial basal medium lacking growth factors and serum or in basal medium supplemented with serum and a cocktail of angiogenic factors. After 72 hours, we observed a decreased expression of miR-21 when endothelial cells were cultured in basal medium complemented with angiogenic factors and serum (Figure 1A). This reduced miR-21 expression did not result from a decrease in cell viability as verified using calcein staining (Figure S1A). In order to identify which factors are responsible for the reduced expression level of miR-21, HUVECs were cultured in basal medium supplemented with the different angiogenic factors added separately, namely serum, bFGF, VEGF, EGF and hydrocortisone. A lower expression of miR-21 was detected in HUVECs after the addition of serum and bFGF to the culture medium. No effects were observed with other growth factors (Figure 1B). The variation of miR-21 expression level in endothelial cells following treatment with growth factors suggests that the expression of this miRNA should be related to endothelial cell quiescence. To test this hypothesis, miR-21 abundance was measured in HUVECs cultured in medium containing angiogenic factors supplemented with the mitogen-activated protein kinase 1/2 (ERK1/2) inhibitor PD-98059 to induce the quiescence of proliferating HUVECs [27]. The addition of PD-98059 in the culture medium induced an increase of miR-21 expression in a dose-dependent manner (Figure 1C). Analysis of the expression of different miRNAs in these conditions revealed that the angiomiRs miR-126 and miR-221 expression levels were also modulated by growth factors addition to the culture medium while miR-16 expression was not affected (Figure S1B).

### miR-21 overexpression impairs angiogenesis *in vitro*

In order to investigate miR-21 function in angiogenesis, we examined the effects of its overexpression in endothelial cell culture assays. Overexpression of miR-21 in HUVECs was obtained by transfecting HUVECs with a pre-miR-21 (Ambion) with a transfection efficiency higher than 90%, as determined using FITC-siRNA (Integrated DNA Technology) (data not shown). The level of miR-21 was increased approximately 40-fold in the presence of the pre-miR-21, as determined by qRT-PCR (Figure S2).

**Figure 2:**
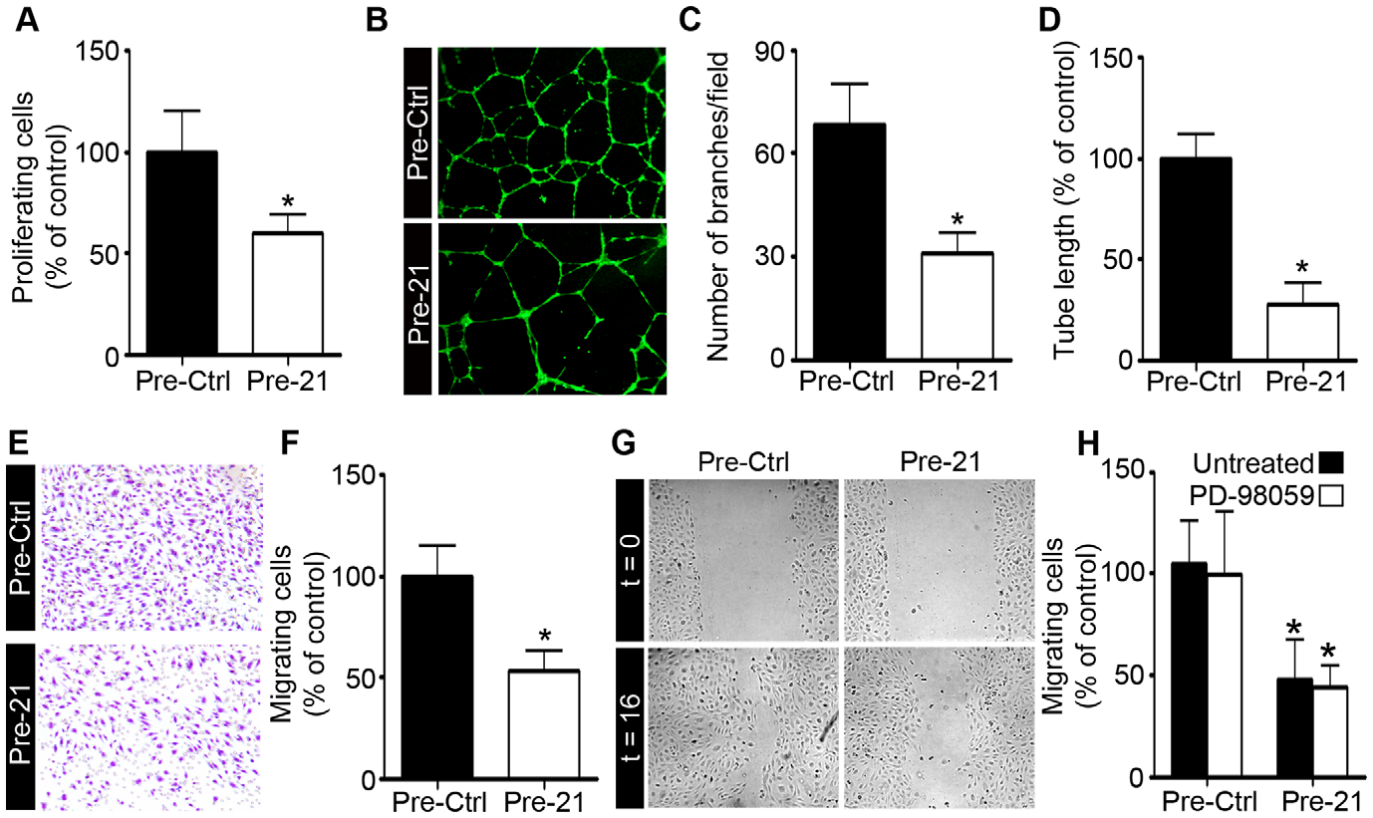
miR-21 overexpression impairs angiogenesis *in vitro.* HUVECs were transfected with a precursor of miR-21 (Pre-21) or with a precursor control (Pre-Ctrl) and were assessed for proliferation, tubulogenesis and migration after 48 h. **A**. Proliferation was determined in transfected HUVECs treated with bFGF (10 ng/ml) and VEGFa (50 ng/ml) by measuring BrdU incorporation (n = 3). **B–D**. Transfected HUVECs were seeded onto Matrigel in EGM-2 and then allowed to form capillary-like structures for 16 h. Living cells were labeled with calcein-AM. Representative figures are shown in (**B**). Branching number (**C**) and tube length (**D**) were quantified with Image J software (n = 5–10 pictures/condition; n = 3 experiments). **E–F**. Transfected HUVECs were allowed to migrate through Boyden chamber inserts for 16 h. The number of cells that migrated to the chamber containing bFGF and serum was counted and quantified with Image J software. **G–H**. Migration of transfected HUVECs in a scratch-wound assay 16 h after treatment with bFGF (10 ng/ml) and VEGFa (50 ng/ml) with or without addition of 10 µM of PD-98059 (n = 6–12 measurements/condition; n = 3 experiments). Data are means with the SD. *p<0.05 versus corresponding control.

Endothelial cell proliferation, migration and organization into tubes are key mechanisms of angiogenesis. In order to investigate the potential of miR-21 as a regulator of angiogenic processes, we first evaluated its ability to affect endothelial cell proliferation. HUVEC proliferation was decreased by 40% upon miR-21 overexpression, as assessed by measuring BrdU incorporation (Figure 2A). No effect was observed after miR-21 overexpression on HUVEC apoptosis (data not shown).

The role of miR-21 as a modulator of endothelial cell organization into tubes was next evaluated. When seeded onto Matrigel^TM^, HUVECs develop into a capillary-like vessel network. Increasing levels of miR-21 in HUVECs reduced vascular network formation, as revealed by a similar decrease in tube branching and total tube length (Figure 2B–D).

In order to investigate whether miR-21 affects endothelial cell migration, another crucial step of angiogenesis, we performed migration assays. A modified Boyden chamber was used to evaluate the ability of HUVECs to migrate to bFGF and serum. A 50% reduction of HUVEC migration was observed in response to miR-21 overexpression (Figure 2E–F). Similar results were observed in a wound closure assay, where increasing miR-21 expression reduced endothelial cell capacity to close the scratch (Figure 2G–H). We also analyzed cell migration in the presence of the proliferation inhibitor PD-98059 to rule out the potential implication of cell proliferation in this assay. Under these conditions, the level of reduced migration (55%) was similar revealing that miR-21 reduces cell migration independently of its effect on proliferation (Figure 2H).

**Figure 3:**
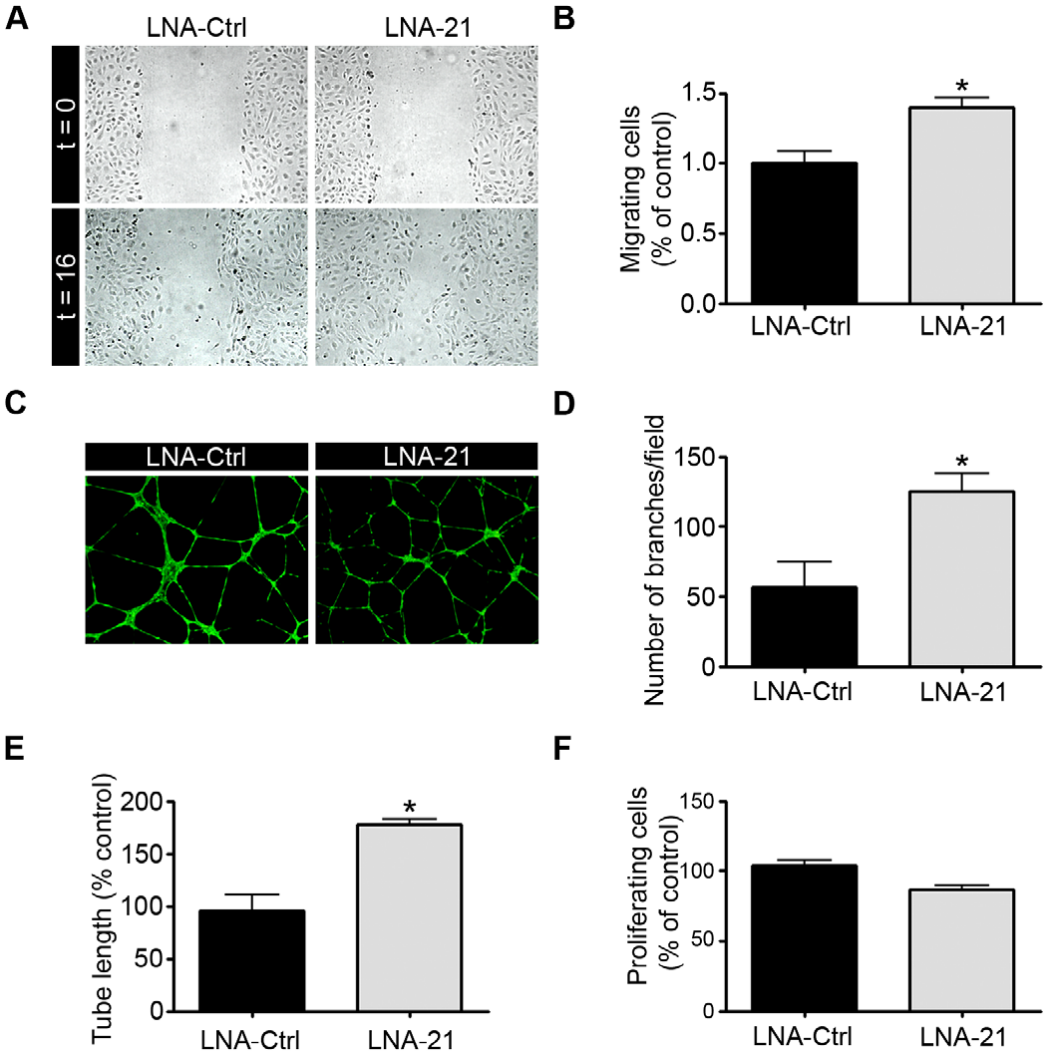
Inhibition of miR-21 impairs angiogenesis *in vitro*. HUVECs were transfected with a LNA-21 or with a LNA control (LNA-Ctrl) and were assessed for migration, tubulogenesis and proliferation after 48 h. **A–B**. Migration of transfected HUVECs in a scratch-wound assay 16 h after treatment with bFGF (10 ng/ml) and VEGFa (50 ng/ml) (n = 6–12 measurements/condition; n = 3 experiments). **C–E**. Transfected HUVECs were seeded onto Matrigel in EGM-2 and then allowed to form capillary-like structures for 16 h. Living cells were labeled with calcein-AM. Representative figures are shown in (**C**). Branching numbers (**D**) and tube length (**E**) were quantified with Image J software (n = 5-10 pictures/condition; n = 3 experiments). Data are means with the SD. *p<0.05. **F**. Proliferation was determined in transfected HUVECs treated with bFGF (10 ng/ml) and VEGFa (50 ng/ml) by measuring BrdU incorporation (n = 3).

To further analyze the role of miR-21 in angiogenesis, we investigated the effect of silencing endogenously expressed miR-21 in endothelial cells. Inhibition of miR-21 expression in HUVECs was achieved by transfecting HUVECs with an anti-miR LNA-21 (Exiqon) and compared with cells transfected with a control LNA (LNA-Ctrl). Silencing of miR-21 in HUVECs was found to increase endothelial cell migration of about 40%, as revealed in a wound closure assay (Figure 3A–B). We next investigated whether endogenous miR-21 could alter the ability of HUVECs to form a vascular network when seeded onto Matrigel^TM^ (Figure 3C). LNA-21 transfected HUVECs increased by 2.3 and 1.9 fold the tube branching number and the total tube length respectively (Figure 3D–E). Proliferation of LNA-21 transfected HUVECs showed a trivial effect (Figure 3F). Taken together, these data indicate that miR-21 affects mainly endothelial cell migration and tubulogenesis.

### miR-21 reduces RhoB expression in endothelial cells

**Figure 4:**
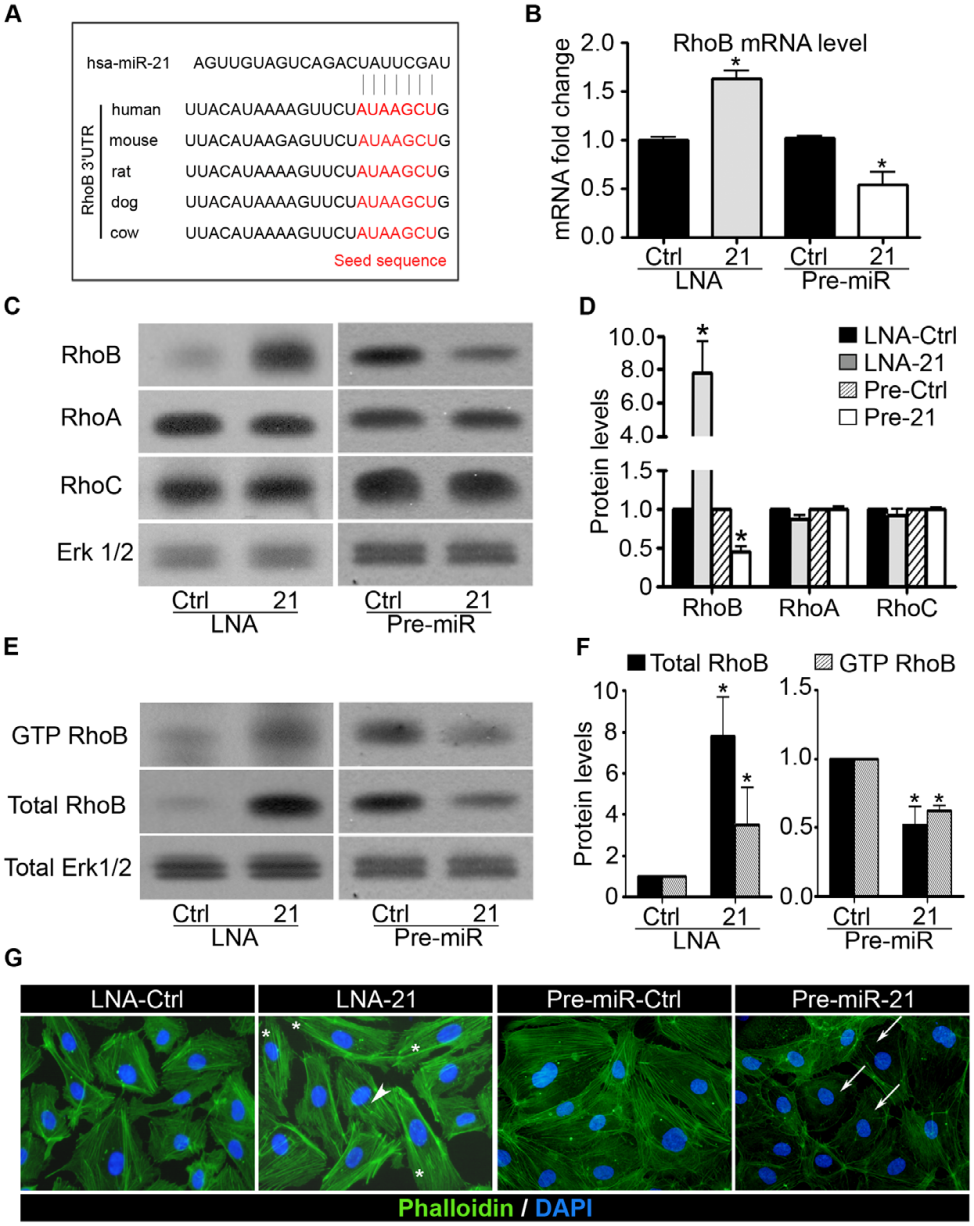
miR-21 reduces RhoB expression and activity in endothelial cells. **A**. Alignment of potential miR-21 binding sites in the 3′UTR of the RhoB mRNA of different species. **B**. HUVECs were transfected with a precursor of miR-21 (Pre-miR-21) or with a precursor control (Pre-miR-Ctrl) and with a LNA-21 or with a LNA control (LNA Ctrl). The RhoB mRNA level was analyzed after 48 h by qRT-PCR. **C–D**. Total protein was extracted from HUVECs 48 h post transfection and RhoA, RhoB and RhoC protein levels were measured by Western blotting. ERK1/2 level was analyzed as an internal control. **D**. Quantification of (**C**). Quantification was performed using ImageJ software. **E–F**. Measurement of GTPase activity of RhoB. **E**. Transfected HUVECs were processed for pull-down assays and Western blot analysis with specific antibody to RhoB. **F**. Quantification of (**E**). Quantification was performed using ImageJ software. **G**. Transfected HUVECs were seeded onto gelatin-coated slides and analyzed for fluorescence labeling by phalloidin-FITC (green). Nuclei were visualized by DAPI staining (blue). White arrows show areas with less actin stress fibers. Asterisks represent some elongated HUVECs. Arrowhead indicates stress fibers in the center of the cell. Pictures are representative of three independent experiments. Data are means with the SD. *p<0.05 versus corresponding control; (n = 3).

In order to identify putative target genes regulated by miR-21 and involved in the control of angiogenesis, we evaluated targets computationally predicted by publicly available algorithms (Targetscan). Using these lists of *in silico* predicted targets, we searched for genes that may play a role in angiogenic processes. Among them we found genes encoding several regulators of endothelial cell functions and vessel growth, such as the RhoGTPase RhoB, Sox7, the transforming growth factor beta receptor II (TGFBRII) and the regulator of ERK activation Sprouty1 (SPRY1). Regulators of cell migration such as the Rho guanine nucleotide exchange factor 12 (ARHGEF12), the myosin phosphatase Rho interacting protein (MPRIP) and vinculin (VCL) are also listed as putative targets of miR-21. Analysis performed by qRT-PCR and/or by Western blotting revealed that *SOX7*, *TGFBRII, ARHGEF12*, *MPRIP* and *VCL* expression was unaffected by miR-21 overexpression in HUVECs while SPRY1 was regulated by miR-21 as revealed by Western blot (Figure S3A-B). Interestingly, the 3′UTR of the *RhoB* mRNA was found to contain one predicted binding site for miR-21 conserved between several species (Figure 4A), which suggest that RhoB might be a direct target of miR-21. Moreover, RhoB was reported very recently to be targeted by miR-21 in hepatocellular carcinoma cell lines. In HUVECs, overexpression of miR-21 significantly decreased the level of *RhoB* mRNA expression (Figure 4B). By contrast, inhibition of miR-21 expression increased *RhoB* mRNA. Regulation of endogenous RhoB expression by pre-miR-21 and LNA-21 was further confirmed at the protein level by Western blotting (Figures 4C-D). RhoB belongs to a family of small GTPases composed of three closely related homologs, A, B and C [28]. In addition, we demonstrated that the regulation of RhoB mediated by miR-21 was specific for this GTPase, since induction or repression of miR-21 had no significant effect on the expression of the other two related RhoGTPases, RhoA and RhoC (Figure 4C-D).

Since RhoB exists under an inactive GDP-bound state and an active GTP-bound state, we tested whether the miR-21 expression level was correlated with the RhoB active state. As measured by a pull-down assay, the increased miR-21 expression resulted in a 40% decreased level of the active form of RhoB. Moreover, miR-21 inhibition using LNA anti-miR resulted in a 5 fold increase of the active RhoB level (Figures 4E–F). Taken together, these results indicate that miR-21 regulates the expression level of active RhoB in endothelial cells.

RhoGTPases are a key determinant in the organization of the actin cytoskeleton and cell shape. RhoB has been reported to induce the assembly of actin stress fibers when overexpressed in endothelial cells [29]. Accordingly, we assessed the ability of miR-21 to affect actin organization. Although some stress fibers were still visible, a reduction in the actin stress fiber network in the center of the cells was observed in cells overexpressing miR-21, as revealed by a phalloidin staining of F-actin (arrows, Figure 4G). Moreover, miR-21 overexpressing cells adopt a more round cell-shape morphology. On the other hand, miR-21 inhibition leads to an increase in stress fiber formation mainly in the center of the cell (arrowhead, Figure 4G). On the other hand, miR-21 silenced-cells adopt a more elongated phenotype (asterisk, Figure 4G). These results highlight the role of miR-21 in actin stress fiber organization.

### RhoB is a direct target of miR-21 involved in angiogenesis

**Figure 5:**
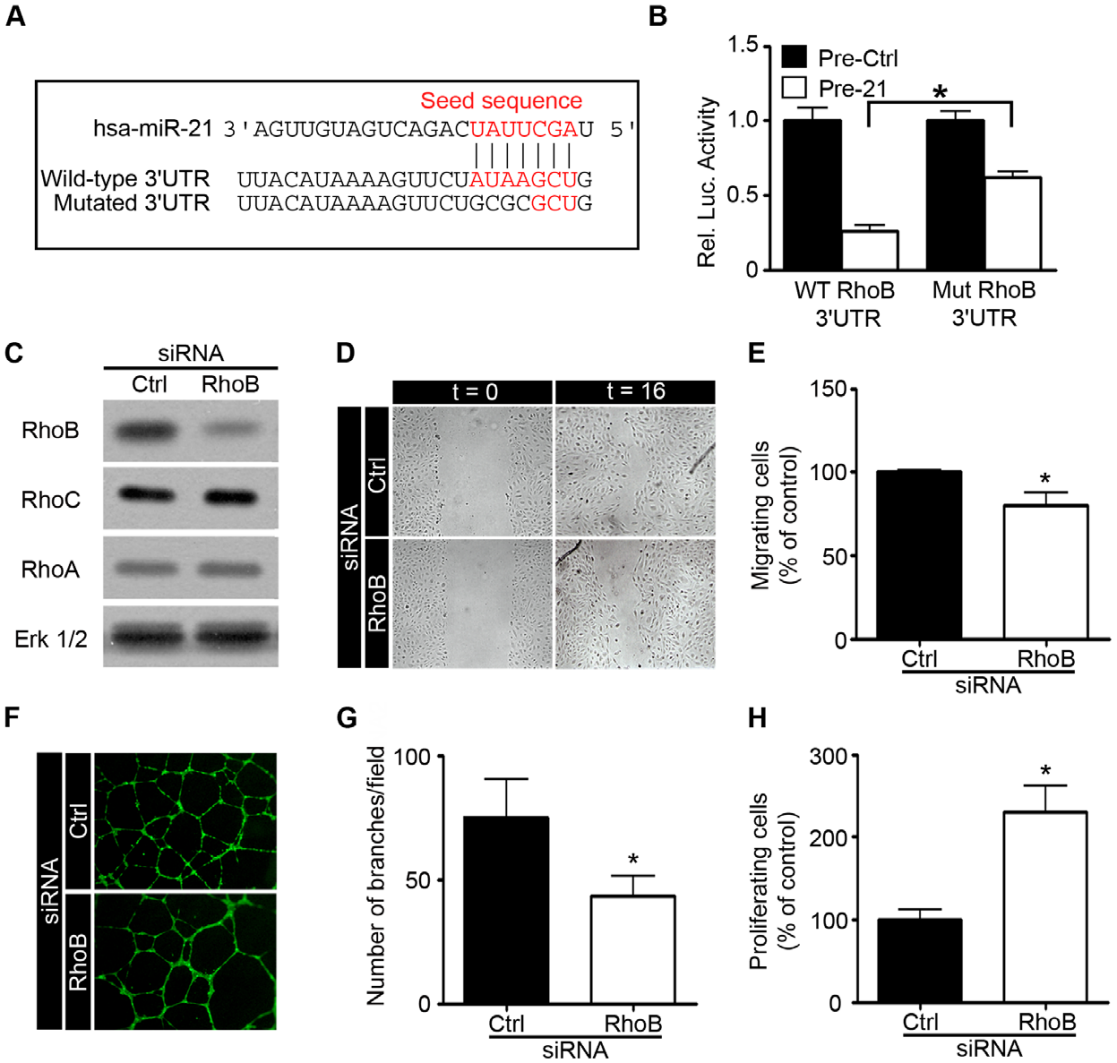
RhoB-silencing alters endothelial cell migration, proliferation and tubulogenesis. **A**. Predicted miR-21 binding site within the RhoB 3′UTR. **B**. The wild-type (WT RhoB 3′UTR) or mutated (Mut RhoB 3′UTR) reporter plasmid was cotransfected into HEK293T cells with a precursor of miR-21 (Pre-21) or with a precursor control (Pre-Ctrl). Luciferase activities were quantified 48 h after transfection as described in the materials and methods section (Luciferase reporter assay and cloning). Renilla luciferase activity was normalized by Firefly luciferase activity. **C**. HUVECs were transfected with non-silencing siRNA (siRNA-Ctrl) or with *RhoB* siRNA and the RhoB protein level was measured after 48 h by Western blotting. The ERK1/2 level was also measured as an internal control. **D–H**. HUVECs were transfected as described above and were assessed for migration, tubulogenesis and proliferation after 48 h. **D–E**. Migration of transfected HUVECs in a scratch-wound assay 16 h after treatment with bFGF (10 ng/ml) and VEGFa (50 ng/ml) (n = 6–12 measurements/condition; n = 3 experiments). **F–G**. Transfected HUVECs were seeded onto Matrigel in EGM-2 and then allowed to form capillary-like structures for 16 h. Living cells were labeled with calcein-AM. Representative figures are shown in (**F**). Branching numbers (**G**) were quantified with Image J software (n = 5–10 pictures/condition; n = 3 experiments). **H**. Proliferation was assayed in transfected HUVEC treated with bFGF (10 ng/ml) and VEGFa (50 ng/ml) by measuring BrdU incorporation (n = 3). Data are means with the SD. *p<0.05 versus corresponding control.

To confirm that RhoB is a direct target of miR-21, we constructed a luciferase reporter vector encoding the complete 3′UTR of RhoB (WT RhoB 3′UTR) as well as a control vector containing mismatches in predicted miR-21 binding site (Mut RhoB 3′UTR) (Figure 5A). Cotransfection of the WT RhoB 3′UTR plasmid and pre-miR-21 in HEK-293T cells resulted in a strong decrease in luciferase activity suggesting that RhoB mRNA is a direct target of miR-21. Importantly, mutations in the sequence targeted by miR-21 in RhoB 3′UTR reduced the observed down-regulation of luciferase activity by pre-miR-21 validating that this predicted binding site is necessary for miR-21 dependent RhoB expression (Figure 5B).

We then investigated whether the effects of miR-21 expression in endothelial cells described above (Figures 2-3) could be mediated by RhoB. If so, inhibition of RhoB expression would have the same effects on endothelial cell functions as overexpressing miR-21. Knockdown of RhoB expression was achieved through the use of siRNAs. First, efficacy and specificity of the siRNA used was verified by Western blotting (Figure 5C). RhoA and RhoC expression levels were unaffected by the transfection with the RhoB-siRNA in HUVECs. The ERK1/2 level was analyzed as an internal control.

We first explored the potential function of RhoB as a modulator of endothelial cell migration. Inhibition of RhoB expression resulted in a significant decrease in HUVEC migration, as revealed by the wound closure assay (Figures 5D–E). Next, the ability of RhoB to interfere with the organization of endothelial cells into capillary-like structures was investigated by a tubulogenesis assay. HUVECs were transfected with siRNAs and seeded onto Matrigel. Silencing of RhoB expression in endothelial cells decreased tube formation, as demonstrated by a 30% reduction in tube branching (Figures 5F–G) compared to the control siRNA. In order to determine whether reducing RhoB expression would affect endothelial cell proliferation, HUVECs were transfected with a RhoB-siRNA or with a control siRNA. A significant increased proliferation was observed in cells where RhoB expression had been silenced, indicating that RhoB-deficient cells had a higher proliferation rate than control cells (Figure 4H). Similar results were obtained using another RhoB-siRNA (data not shown).

To confirm that the reduction in cell migration mediated by miR-21 is due to RhoB inhibition, we performed miR-21 and RhoB knockdown, individually or in combination. Inhibition of miR-21 alone (cotransfection with control siRNA and LNA-21) led to an increased RhoB expression and to an increased migration (Figure S4A–B). Conversely, silencing of RhoB alone (cotransfection with control LNA and RhoB-siRNA) led to a decreased of RhoB expression and to a reduced migration. Silencing of RhoB combined with the silencing of miR-21 restored the level of RhoB expression as well as the migration rate (Figure S4A–B). Altogether these data confirmed the miR-21-dependent regulation of RhoB and its implication in the migrating properties of HUVECs.

### miR-21 inhibits pathological angiogenesis in a mouse model of choroidal neovascularization

**Figure 6:**
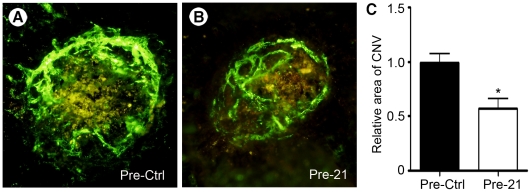
miR-21 inhibits pathological angiogenesis in a mouse model of choroidal neovascularization. Adult C57BL/6 mice were subjected to laser-induced Bruch's membrane rupture in each eye, and then intravitreally injected with pre-miR-21 (Pre-21) or a control molecule (Pre-Ctrl). Seven days after Bruch's membrane rupture at 4 locations, the mice were injected with fluorescein-labeled dextran and the eyes were removed and mounted for microscopic analysis. Representative pictures are shown in (**A–B**). **C**. The choroidal neovascularization area at the Bruch's membrane rupture sites was quantified with Image J software. Relative choroidal neovascularization is plotted on the graph. Pictures are representative of three independent experiments. Optical magnification 10x (n≥8 eyes/experiment; n = 3 experiments). Data are means with the SD. *p<0.05.

In order to evaluate the impact of miR-21 *in vivo* in pathological angiogenesis, we used a laser-induced murine model of choroidal neovascularization (CNV), which mimics the age-related macular degeneration (AMD) pathology [30]. Intravitreal injection of pre-miR-21 or the control molecule was performed just after laser burns. Neovascularization in the lesion was assessed seven days later by epifluorescence microscopy on choroid flat-mounts following intravenous injection of FITC-dextran (Figure 6A–B). Isolectin-B4 staining of laser-burned eyes showed that FITC-dextran is localized in some, but not all, blood vessels (Figure S5A–C). Quantification of the FITC-perfused area revealed a marked reduction in blood vessel density in animals treated with pre-miR-21 (Figure 6C). These results indicate the therapeutic efficacy of miR-21 induction in pathological angiogenesis settings.

## Discussion

Several pathologies are associated with excessive angiogenesis such as cancer, macular degeneration and diabetic retinopathy. To date, few angiogenic inhibitors have been used in therapy and these have failed to produce an enduring clinical response in most patients. Their low efficacy is notably due to the appearance of resistance mechanisms, as these inhibitors are specific for a single angiogenic receptor [31], [32]. Hence, the ability of one miRNA to target multiple mRNAs renders them very attractive. Manipulating angiomiRs in settings of pathological vascularization thus represents a new therapeutic approach. To date, only few miRNAs have been found to be involved in angiogenesis regulation [17].

From the present study, miR-21 emerges as a new angiomiR which negatively regulates angiogenesis. Using different *in vitro* angiogenic assays, we demonstrated that miR-21 overexpression affects endothelial cell proliferation, migration and organization into tubes. miR-21 was also found to modify actin cytoskeleton organization. The organization of actin into stress fibers is known to play an essential role in promoting cell migration [33]. The decrease in endothelial cell migration observed upon miR-21 induction may thus be due to reduced stress fiber formation.

In line with its function as a negative modulator of angiogenesis, we monitored a reduced expression of miR-21 upon the addition of angiogenic factors to the culture medium. Our results showed that bFGF and serum acted as inhibitors of miR-21 expression, as treatment with these molecules was found to decrease miR-21 expression. The increased expression of miR-21 in quiescent HUVECs suggests that miR-21 could be implicated in the maintenance of the quiescent vascular endothelium. Interestingly, the well-known angiogenic miRNA miR-126 followed a similar pattern highlighting the important role of miRNA in vascular homeostasis control. The results published by Würdinger *et al.*, who described a 5-fold reduction in miR-21 and miR-126 levels in human brain microvascular endothelial cells (HBMVECs) cocultured with glioma cells, used to stimulate angiogenesis [34], supports our results. Investigation of the level of miR-21 and miR-126 in proliferating versus quiescent blood vessels could further help to better understand the role of these miRNAs in vessel homeostasis.

By affecting key steps of endothelium remodeling, modulation of miR-21 should provide new strategies to control pathological angiogenesis. Age-related macular degeneration (AMD), a disease characterized by an excessive choroidal vascularization (CNV), is the most frequent cause of blindness in western industrialized countries. In a mouse model of CNV, which mimics what happens during AMD, we demonstrated that miR-21 overexpression reduces neovascularization. These findings suggest the potential use of miR-21 as a therapeutic tool for treating diseases associated with excessive angiogenesis. Further studies are still needed to investigate more precisely the relevance of miR-21 therapeutic use in other angiogenesis-related diseases such as tumor development.

The antiangiogenic role of miR-21 observed in our study may be surprising regarding its implication in tumor progression. Indeed, miR-21 is overexpressed in the vast majority of cancers [6] and its expression seems to favor tumor growth and invasion. Inhibition of miR-21 expression has been demonstrated to increase apoptosis in cultured glioblastoma cells [11], to reduce breast cancer MCF7 cell growth *in vitro* and to reduce tumor growth in a xenograft mouse model [35]. In addition, decreased tumor cell proliferation, migration and invasion have been found upon miR-21 silencing in hepatocellular cells [36]. Reduction in miR-21 expression has also been associated with a reduction in metastasis in breast and colorectal cancer [37], [38]. However, in our experiments, miR-21 expression induced defects in angiogenesis processes, as demonstrated by a reduction in endothelial cell tubulogenesis and migration, without any effect on apoptosis (data not shown). One explanation of these opposing roles of miR-21 in tumor cells and the endothelium compartment could be that miR-21 targets different genes in different cell types. Previous studies have shown that the differential expression or availability of the targets between different tissues may direct an miRNA to affect one specific set of targets [39]. For example, opposing effects on the proliferation of cancer cells and endothelial cells have already been reported for miR-221/222. Indeed, whereas high expression of miR-221/222 blocks angiogenesis and proliferation in endothelial cells via c-kit [18], it promotes proliferation in cancer cells by targeting the cell cycle regulator p27 [40]. These data suggest that regulation of proliferation by miR-221 and miR-222 is specific to the cell type. We thus hypothesize that similar processes occur with miR-21.

In order to elaborate a putative mechanism for miR-21 to inhibit angiogenesis, we focused on the identification of the targets of this miRNA. Taken together, our results support a role for the RhoGTPase RhoB in the negative function of miR-21 in angiogenesis. Indeed, RhoB expression and activity were found to be reduced upon miR-21 expression. RhoGTPases are a family of 20 small G proteins notably regulating actin cytoskeleton, cell polarity, vesicular trafficking and gene expression [41]. A growing amount of evidence has also implicated the Rho-proteins as key regulators of angiogenesis through the modulation of various steps of this process, including vascular permeability, extracellular matrix remodeling, migration and proliferation [42]. The three best-studied members of the Rho family are RhoA, Rac1 and cdc42. RhoB has essentially been described in tumor cells where it seems to act as a tumor suppressor gene [43]. Few studies have described its role in endothelial cells. One group reported a retarded vascular development in the retina of mice deficient in RhoB [44]. In cultured endothelial cells, RhoB has been associated with an increased apoptosis and a failure in tube formation upon RhoB depletion [44]. Bacterial toxins have also been shown to inhibit endothelial cell migration by inhibiting the three related RhoGTPases RhoA, RhoB and RhoC [45]. In line with these data, our knockdown experiments using RhoB specific siRNAs indicated a decreased migration capacity and a reduced organization into tubular structures in endothelial cells. These effects do not result from defects in apoptosis (data not shown). The reduction in these processes observed upon miR-21 induction may thus be explained at least partially, by reduced RhoB expression and activity caused by miR-21.

Interestingly, the RhoB mRNA contains one putative conserved binding site for miR-21 in the 3′UTR, suggesting that RhoB is a direct target of miR-21. The conservation of this site during the evolution suggests that this target may be of functional relevance. Moreover, a very recently published study demonstrated an interaction between the 3′UTR of RhoB and miR-21 in a hepatocellular carcinoma cell line [26]. All these data strongly support the fact that, in endothelial cells, miR-21 also represses RhoB expression by directly targeting its 3′UTR.

Both overexpression and silencing of miR-21 indicate that miR-21 regulates migration and tubulogenesis. Since silencing of endogenous miR-21 did not confirm the implication of miR-21 in cell proliferation, we believe that the reduced proliferation observed in Pre-miR-21 overexpression is physiologically irrelevant. Our results with RhoB silencing support this interpretation. RhoB silencing decreased migration and tubulogenesis. Combined with the double knockdown experiments, these results strongly highlight RhoB as a key target of miR-21-mediated angiogenesis.

However, additional target genes are likely to participate to miR-21 antiangiogenic effects. Among the putative targets, we selected SOX7, MPRIP, SPRY1, ARHGEF12, TGFBRII and VCL that might be involved in regulation of angiogenesis. Except Spry1, whose expression is down-regulated at the protein level, all other tested genes were unaffected by miR-21. SPRY1 was shown to inhibit ERK signaling and we recently showed that SPRY1 is a negative regulator of angiogenesis [24]. SPRY1 role in miR-21 antiangiogenic response is thus conflicting and further investigations would clarify its implication in miR-21 response.

In summary, the findings of the present study identify miR-21 as a novel member of the angiomiR family, acting by negatively regulating key angiogenic processes such as endothelial cell migration and tubulogenesis. Our research for relevant target genes for miR-21 leads us to propose a role for RhoB. Moreover, the significant reduction in angiogenesis observed in an *in vivo* mouse model of CNV after miR-21 overexpression presents miR-21 as a therapeutic tool for treating aberrant angiogenesis.

## Materials and Methods

### Mice

All animal experiment protocols were approved by the Institutional Ethics Committee of the University of Liège and in compliance with the ARVO Statement for the Use of Animals in Ophthalmic and Vision Research (permit number 674).

### Cell culture

Human umbilical vein endothelial cells (HUVECs) were isolated as previously described [46]. Briefly, umbilical veins were washed with PBS, filled with trypsin solution (0.05% trypsin (Difco); 0.2% EDTA; PBS; pH 7.6), clamped, and incubated in a 37°C water bath for 20 minutes. The umbilical vein was washed with PBS and the eluate was centrifuged to harvest cells. HUVECs were seeded onto gelatin-coated culture dishes and cultured in EGM-2 medium (EGM-2 BulletKit medium (CC-3162, Lonza) supplemented with 5% fetal bovine serum (FBS)). PD-98059 (Calbiochem) was used at 10 µM in functional assay and at 5 to 25 µM for dose response experiment.

### Quantitative miRNA expression analysis by Taqman assay

Total RNAs were extracted with the miRNeasy kit (Qiagen). Taqman methods were used to assess miRNA expression. Briefly, 10 ng RNA was reverse transcribed to cDNA with the Taqman microRNA Reverse Transcription kit and the Taqman microRNA assay stem loop primers (Applied Biosystems). Resulting cDNAs were used for quantitative real-time PCR using Taqman microRNA assay and Taqman universal PCR master mix reagents (Applied Biosystems). Thermal cycling was performed on an ABI Prism 7900 HT Sequence Detection System (Applied Biosystems). For all reactions, no-template controls were run, and random RNA preparations were also subjected to sham reverse transcription to check for the absence of genomic DNA amplification. The relative miRNA level was calculated by the 2^−ΔΔCt^ method [47] and normalized to RNU-44 (Applied Biosystems).

### *In vitro* modulation of miRNA-21 and RhoB levels

Modulation of the miR-21 level was performed with modified oligonucleotides provided by Ambion. For overexpression of miR-21, HUVECs were transfected with 5 µl of 10 µM pre-miR-21 or with control pre-miR oligonucleotides. For inhibition of miR-21, HUVECs were transfected with 15 nM of LNA-21 or control LNA (Exiqon). For inhibition of RhoB expression, HUVECs were transfected with a RhoB-siRNA at a final concentration of 20 nM or with a control siRNA. HUVECs were transfected with siRNA, LNA or pre-miR molecules with Dharmafect-4 reagent (Dharmacon Research) according to the manufacturer's instructions. Briefly, 1 µl Dharmafect-4 (Dharmacon Research) was mixed with 299 µl RPMI1640 (Lonza) and incubated for 10 minutes at room temperature. siRNA, LNA or pre-miR molecules were mixed with 295 µl RPMI1640 (Lonza) and incubated for 10 minutes at room temperature. siRNA, LNA or pre-miR mix was added to the Dharmafect-4 mix and incubated for 20 min at room temperature before pouring into a 25-cm^2^ flask. Cells were then seeded into the flask (7×10^5^ cells in 1.4 ml EGM-2 medium) and incubated for 16 h at 37°C. The medium was then replaced with 3 ml EGM-2 medium and the cells incubated at 37°C for 24 h or more.

### Boyden chamber migration assay

HUVECs were transfected as described above with pre-miR oligonucleotides for 48 h and divided into eight-micrometer 24-well Boyden chambers (Transwell; Costar Corp) used for cell migration assays. Both sides of the membrane were coated overnight with 0.005% gelatin. The lower chamber was filled with 600 µl EBM-2 medium (Lonza) containing 1% BSA, 1% FBS, and 10 ng/ml recombinant bFGF (Promega Corp). Transfected HUVECs were placed in 300 µl of 0.1% BSA/EBM-2 in the upper chamber and allowed to migrate for 16 h at 37°C. After fixation, cells stained with 4% Giemsa were counted on the lower side of the membrane. Cell counting was performed with an ImageJ (http://rsbweb.nih.gov/ij/) macro relying on color thresholding in the RGB color space, followed by connected component labeling with the “Analyze Particles” function using size and circularity criteria. The same set of parameters was used for all experiments and detection masks were produced and double-checked by visual examination.

### Scratch wound migration assay

HUVECs transfected with siRNA, LNA or pre-miR as described above were trypsinized 48 h post-transfection. Following this, 5×10^4^ cells were seeded into a 48-well plate in 350 µl EGM-2 medium (Lonza) and incubated for 24 h to reach confluence. Using a tip, a wound was made in the monolayer (at time 0). The cells were then washed with PBS and incubated with EBM-2 medium containing 10 ng/ml recombinant bFGF (Promega Corp.) and 50 ng/ml recombinant VEGFa (RELIATech GmbH) for 16 h. The distance between the two sides of the wound was measured with a graduated ocular lens coupled with an Olympus CKX41 microscope (Olympus). The distance between the two sides of the wound after 16 h of migration was substracted from the distance at time 0 and represented on a graph.

### Capillary network formation on a Matrigel matrix

The ability of RhoB siRNA-, LNA-21- or pre-miR-21-transfected HUVECs to form capillary networks was evaluated in a Matrigel^TM^ angiogenesis assay. Briefly, 8750 HUVECs transfected as described above were plated in a 96-well plate pre-coated with 35 µl Matrigel per well (BD Biosciences) 48 h post-transfection. The cells were incubated in EGM-2 medium for 16 h (Lonza). In order to visualize vessels under a fluorescence microscope, the cells were incubated with calcein-AM (2 µM) for 20 min. Pictures were taken with an Olympus fluorescence microscope (Olympus). Quantitative analysis of network structure was performed with ImageJ software (http://rsbweb.nih.gov/ij/) by counting the number of intersections in the network and measuring the total length of the structures.

### Analysis of cell proliferation

HUVECs were transfected as described above with siRNA, LNA or pre-miR oligonucleotides for 48 h. Following this, transfected cells were plated in 96-well culture plates at a density of 1500 cells per well in EGM-2 medium (Lonza) and allowed to adhere for 16 h. The medium was washed with PBS and replaced with EBM-2 medium supplemented with recombinant bFGF (10 ng/ml) (Promega Corp.) and recombinant VEGFa (50 ng/ml) (RELIATech GmbH). 16 hours later, BrdU was added and the culture was incubated for 8 hours. BrdU incorporation was measured with the Cell Proliferation ELISA BrdU (chemiluminescence) kit (Roche Applied Sciences) according to the manufacturer's protocol.

### qRT-PCR analysis of gene expression

RNAs were extracted with the miRNeasy kit (Qiagen) according to the manufacturer's protocol. cDNA synthesis was performed with 1 µg total RNA and the iScript cDNA Synthesis Kit (Biorad) according to the manufacturer's instructions. Resulting cDNAs were used for quantitative real-time PCR using the SYBR green method (Roche Applied Sciences). Thermal cycling was performed on an ABI Prism 7900 HT Sequence Detection System (Applied Biosystems). For all reactions, no-template controls were run, and random RNA preparations were also subjected to sham reverse transcription to check for the absence of genomic DNA amplification. The relative transcript level of each gene was obtained by the 2^−ΔΔCt^ method [47] and normalized with respect to the housekeeping gene cyclophilin a (PPIA) or glyceraldehyde-3-phosphate dehydrogenase (GAPDH). Primers were designed with the Primer Express software (Applied Biosystems) and selected so as to span exon-exon junctions to avoid detection of genomic DNA (see Table S1 – List of primers used in quantitative RT-PCR experiments).

### Western blotting

Cytoplasmic cell lysate was resolved by SDS-PAGE and transferred to a polyvinylidene fluoride membrane (Millipore Corp.). Blots were blocked for 1 h at room temperature with 8% milk in Tris-buffered saline with 0.1% Tween 20 and probed overnight at 4°C with anti-RhoB, RhoA or RhoC primary antibody (RhoA sc-418; RhoB sc-180, RhoC sc-26480 Santa Cruz Biotechnology) (or for 1 h at room temperature for anti-ERK1,2 M-5670, VCL V-9131, Sigma; Sox7 sc-134024, Santa Cruz Biotechnology; MPRIP ab56164, Abcam and 2 h at 37°C for SPRY1 sc-380048 Santa Cruz Biotechnology). After three washes with Tris-buffered saline containing 0.1% Tween 20, antigen-antibody complexes were detected with peroxidase-conjugated secondary antibody and a fluoro-chemiluminescent system (GE Healthcare).

### Cytoskeleton labeling

HUVECs were transfected with pre-miR or LNA molecules as described above. 24 h later, cells were harvested and 3×10^4^ cells were seeded onto glass coverslips pre-coated with gelatin (2 g/l), in 24-well plates containing 500 µl of EGM-2 medium (Lonza, Verviers, Belgium). 24 h later, medium was removed and cells were washed twice. For fibrillar actin labeling, HUVECs were fixed with 1% paraformaldehyde in PBS for 30 min and permeabilized with 0.2% Triton X-100 in PBS for 5 min. The samples were blocked with 0.2% bovine serum albumin in PBS for 30 min and incubated with 5 µg/ml of phalloidin-FITC and with 2 µg/ml of 4′,6-diamidino-2-phenylindole for 30 min. Stained cells were washed twice in PBS and mounted using Vectashield mounting medium (Vector Laboratories, Burlingame, CA). Pictures were obtained using a Nikon Eclipse 90i epifluorescent microscope coupled to a digital camera (Nikon Belux, Brussels, Belgium).

### GTPase assay

HUVECs were transfected with pre-miR or LNA molecules, as previously described, for 48 h. The GTPase assay was carried out as previously described [48]. Briefly, cells were chilled on ice and lysed in ice-cold buffer containing 0.5% Triton X-100, 25 mM Hepes pH 7.3, 150 mM NaCl, 4% glycerol, 5 mM MgCl2, 0.5 mM EGTA, 10 mM NaF, 20 mM β-glycerophosphate, 5 mM DTT and a tablet of protease inhibitor cocktail (Roche Applied Sciences). Lysates were centrifuged for 5 min at 14 000 g. Supernatants were immediately frozen in liquid nitrogen and stored at −80°C. An aliquot of each supernatant collected before freezing was denatured in SDS-PAGE lysis buffer to measure the total RhoGTPase content by Western blotting. For the pull-down assay, supernatants were incubated for 30 min with 30 µg of GST-RBD fusion protein containing GST fused to the Rho-binding region of rhotekin affinity, linked to glutathione-Sepharose beads. The beads were washed 4 times in lysis buffer and boiled in 60 µl of SDS-PAGE lysis buffer.

### Luciferase reporter assay and cloning

Full length RhoB 3′UTR was amplified by PCR using the following primers: forward CTCGAGACTGCTGCAAGGTGCTATGAGG; reverse GCGGCCGCCTCGCCTAGGAGA ATATCTCCC; and cloned in the PsiCheck-2 vector (Promega) using XhoI, HindIII restriction sites. Mutagenesis of the seed sequence was performed using the QuikChange® Site-Directed Mutagenesis Kit (Stratagene) following manufacturers' instructions. For measuring luciferase activity, 175000 HEK293T cells were transfected in 24-well plate with 30 pmol of Pre-miR-control or Pre-miR-21 oligonucleotides as described above. The day after, wild-type or mutated RhoB-Psicheck-2 was transfected using JET-PEI (Polyplus transfection) according to manufacturers' instructions and incubated for 48 h. The activity of Renilla and Firefly Luciferase was then assessed using the Dual-Luciferase® Reporter Assay System (Promega) following manufacturer protocol.

### *In vivo* choroidal neovascularization experiment

The two-month-old C57BL/6 mice (five mice in each group) used in this study were maintained in a 12 hours light–12 hours dark cycle and had free access to food and water. All animal experiments were performed in compliance with the ARVO Statement for the Use of Animals in Ophthalmic and Vision Research. Choroidal neovascularization (CNV) was induced in mice by laser burns as described previously [49]. Mice were anesthetized with an *ip* injection of Avertin. Both pupils were dilated with 1% tropicamide; four burns were delivered (usually at the 3, 6, 9, and 12 o'clock positions around the optic disc) using a green argon laser (532 nm; 50 mm diameter spot size; 0.05 sec duration; 400 mW). The eyes were locally anesthetized with 4 mg/ml Oxybuprocaini HCl (Unicaïne 0.4% - Théa), with this being immediately followed by intravitreal injection of 2 µl control pre-miR or pre-miR-21 at 5 µM. After this, 3 mg/g ofloxacin ointment (Trafloxal® - Dr. Mann Pharma) was applied to the eye. Seven days later, the mice were injected (*i.v.*) with 200 µl fluorescein isothiocyanate (FITC)-conjugated dextran (Mr: 2×10^3^, Sigma-Aldrich) in PBS, pH 7.4 just before exsanguination. Afterward, the eyes were removed and fixed in 1% paraformaldehyde, pH 7.4 for 1 h at room temperature. The retinas were discarded and the choroid was prepared in Vectashield medium (Vector Laboratories) for epifluorescence microscopy (Nikon Belux). Quantification was carried out by measuring the total vessel fluorescence surface (ImageJ software http://rsbweb.nih.gov/ij/, NIH) as described in figure S6A-D. Seven days after choroid lesions appeared at thirty different locations, total RNA extraction was performed for miRNA quantification as described above.

### Isolectin-B4 staining of fluorescein-labeled eyes

Paraformaldehyde 1% fixed eyes were trypsinised (0,025%) at 37°C during 25 min. Eyes were washed and blocked in milk 1.5% during 30 min at room temperature and then incubated in PBS milk 10%, containing IB4-Biotinylated antibody for 2 h at 37°C (Griffonia Simplicifolia isolectin-B4 biotinylated antibody, I-21414, Molecular Probes). Eyes were washed with PBS and incubated with Streptavidin-Cy3 antibody 30 min at room temperature, washed in PBS and mounted with Vectashield (Vector Laboratories). Pictures were obtained using an AH3-RFCA epifluorescence microscope coupled to a digital camera (Olympus).

### Quantifications and statistical analysis

Analyses for statistical significance (Unpaired t-test) were carried out with Prism 4.0 software (GraphPad Software, San Diego, CA, USA). Statistical significance was set at *P*<0.05. All data are expressed as means ± SD.

## Supporting Information

Figure S1 **HUVEC viability in basal medium and miRNA expression in medium containing or not growth factors.** **A**. HUVECs were cultured in basal medium (EBM) and were analyzed for fluorescence by calcein staining (2 µM) after 72 hours. **B**. HUVECs were cultured in EBM, in EGM or in EGM supplemented with 10 µM of PD-98059 for 72 hours. Expression of miR-21, miR-126, miR-16 and miR-221 was quantified by qRT-PCR. The data were normalized to RNU-44 and converted using the formula 2^–ΔΔCt^ (relative expression). Data are means with the SD. *p<0.05 versus corresponding control; (n = 3).
(TIF)

Figure S2 **miR-21 expression following precursor molecule transfections.** HUVECs were transfected with a precursor of miR-21 (Pre-21) or with a precursor control (Pre-Ctrl) for 72 h. miR-21 expression was quantified by qRT-PCR. The data were normalized with respect to RNU-44 and converted using the formula 2^–ΔΔCt^ (relative expression). Data are means with the SD. *p<0.05 versus corresponding control; (n = 3).
(TIF)

Figure S3 **Regulation of** ***SOX7*** **,** ***TGFBRII*** **,** ***ARHGEF12*** **,** ***MPRIP*** **,** ***VCL*** **and** ***SPRY1*** **expression by miR-21.** **A**. HUVECs were transfected with a precursor of miR-21 (Pre-21) or with a precursor control (Pre-Ctrl) for 48 h. *SOX7, SPRY1*, *TGFBRII*, *ARHGEF12*, *MPRIP* and *VCL* expression was analyzed by qRT-PCR. The data were normalized with respect to PPIA and converted using the formula 2^–ΔΔCt^ (relative expression). Data are means with the SD. *p<0.05 versus corresponding control; (n = 3). **B**. Total protein was extracted from HUVECs 48 h post transfection and MPRIP, SOX7, SPRY1 and VCL protein levels were measured by Western blotting. β-tubulin was analyzed as an internal control (representative of 3 independent experiments).
(TIF)

Figure S4 **Double knockdown of miR-21 and RhoB restore RhoB expression and HUVECs migration.** HUVECs were transfected with a LNA-21 or with a LNA control (LNA-Ctrl). Twenty-four hours later, HUVECs were transfected again with non-silencing siRNA (Control-siRNA) or with *RhoB* siRNA (RhoB siRNA). **A**. The RhoB mRNA level was analyzed 24 h after transfection with siRNAs by qRT-PCR. The data were normalized to GAPDH and converted using the formula 2^–ΔΔCt^ (relative expression). **B**. Transfected HUVECs were assessed 48 h later for migration in a scratch-wound assay 16 h after treatment with bFGF (10 ng/ml) and VEGFa (50 ng/ml) (n = 6-12 measurements/condition; n = 3 experiments). Data are means with the SD. *p<0.05 versus corresponding control.
(TIF)

Figure S5 **Staining of the choroidal neovascularized blood vessels.** Adult C57BL/6 mice were subjected to laser-induced Bruch's membrane rupture in each eye, and then intravitreally injected with pre-miR-21 or a control molecule. Seven days after Bruch's membrane rupture at 4 locations, the mice were injected with fluorescein-labeled dextran and the eyes were removed and mounted for microscopic analysis. **A**. Representative picture. **B**. Isolectin-B4 staining of fluorescein-labeled eyes. **C**. Overlay of (A-B). Arrows indicate fluorescein- and isolectin-B4- labeled blood vessel. Arrowheads indicate isolectin-B4-labeled blood vessel not perfused with fluorescein.
(TIF)

Figure S6 **Choroidal neovascularization quantification.** Adult C57BL/6 mice were subjected to laser-induced Bruch's membrane rupture in each eye, and then intravitreally injected with pre-miR-21 or a control molecule. Seven days after Bruch's membrane rupture at 4 locations, the mice were injected with fluorescein-labeled dextran and the eyes were removed and mounted for microscopic analysis. **A-D**. Fluorescein-labeled blood vessels quantification. **A**. Original Red-Green-Blue color picture. **B**. Selection of the blood vessels area using ImageJ erases the background from the original picture. **C**. Extraction of the green component. **D**. Quantification using ImageJ of the pixels (shown in red in the picture) after application of the threshold to mainly select blood vessels structures.
(TIF)

Table S1 **List of primers used in quantitative RT-PCR.** Sequences of all primers used in qRT-PCR experiments are listed.
(DOC)
