# Supplementary figures and images for: MicroRNA-21 Exhibits Antiangiogenic Function by Targeting RhoB Expression in Endothelial Cells
Source: PLoS One. 2011 Feb 10;6(2):e16979. doi: 10.1371/journal.pone.0016979 (PMC3037403; doi:10.1371/journal.pone.0016979)

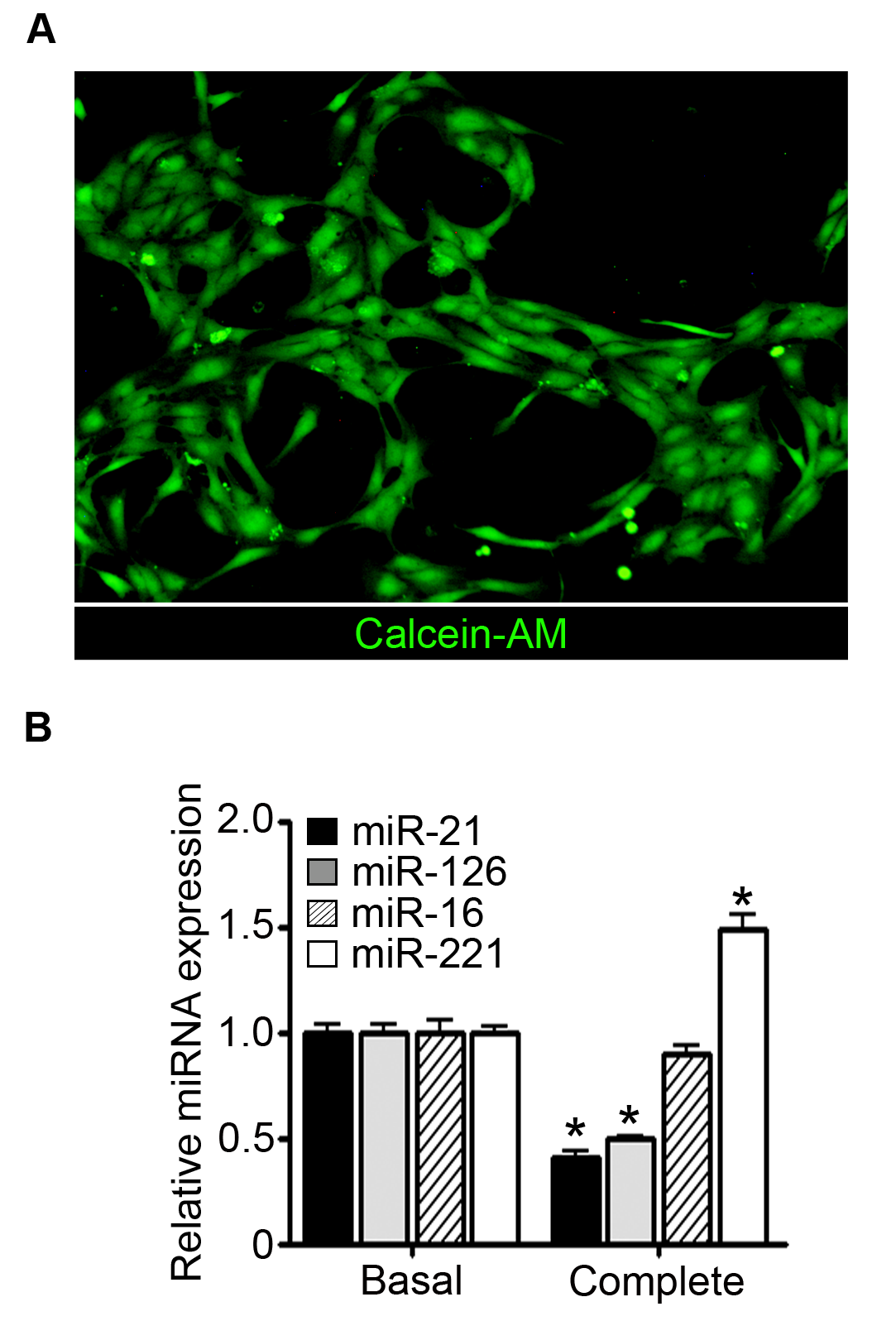

Supplement: Figure S1 — HUVEC viability in basal medium and miRNA expression in medium containing or not growth factors. A. HUVECs were cultured in basal medium (EBM) and were analyzed for fluorescence by calcein staining (2 µM) after 72 hours. B. HUVECs were cultured in EBM, in EGM or in EGM supplemented with 10 µM of PD-98059 for 72 hours. Expression of miR-21, miR-126, miR-16 and miR-221 was quantified by qRT-PCR. The data were normalized to RNU-44 and converted using the formula 2–ΔΔCt (relative expression). Data are means with the SD. *p<0.05 versus corresponding control; (n = 3). (TIF) [file pone.0016979.s001.tif]

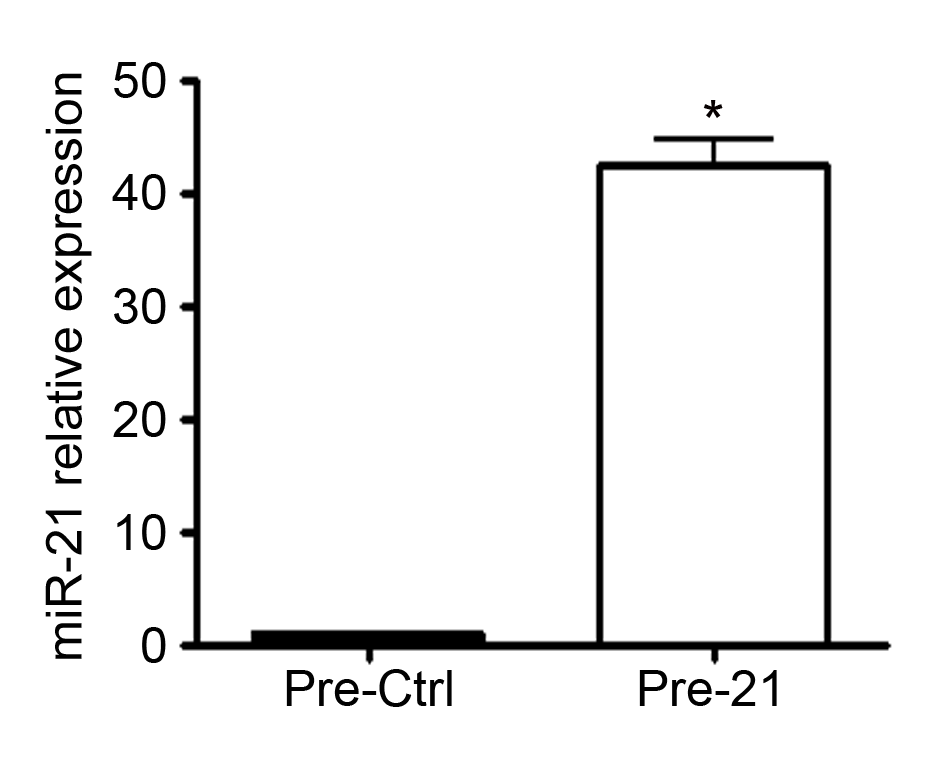

Supplement: Figure S2 — miR-21 expression following precursor molecule transfections. HUVECs were transfected with a precursor of miR-21 (Pre-21) or with a precursor control (Pre-Ctrl) for 72 h. miR-21 expression was quantified by qRT-PCR. The data were normalized with respect to RNU-44 and converted using the formula 2–ΔΔCt (relative expression). Data are means with the SD. *p<0.05 versus corresponding control; (n = 3). (TIF) [file pone.0016979.s002.tif]

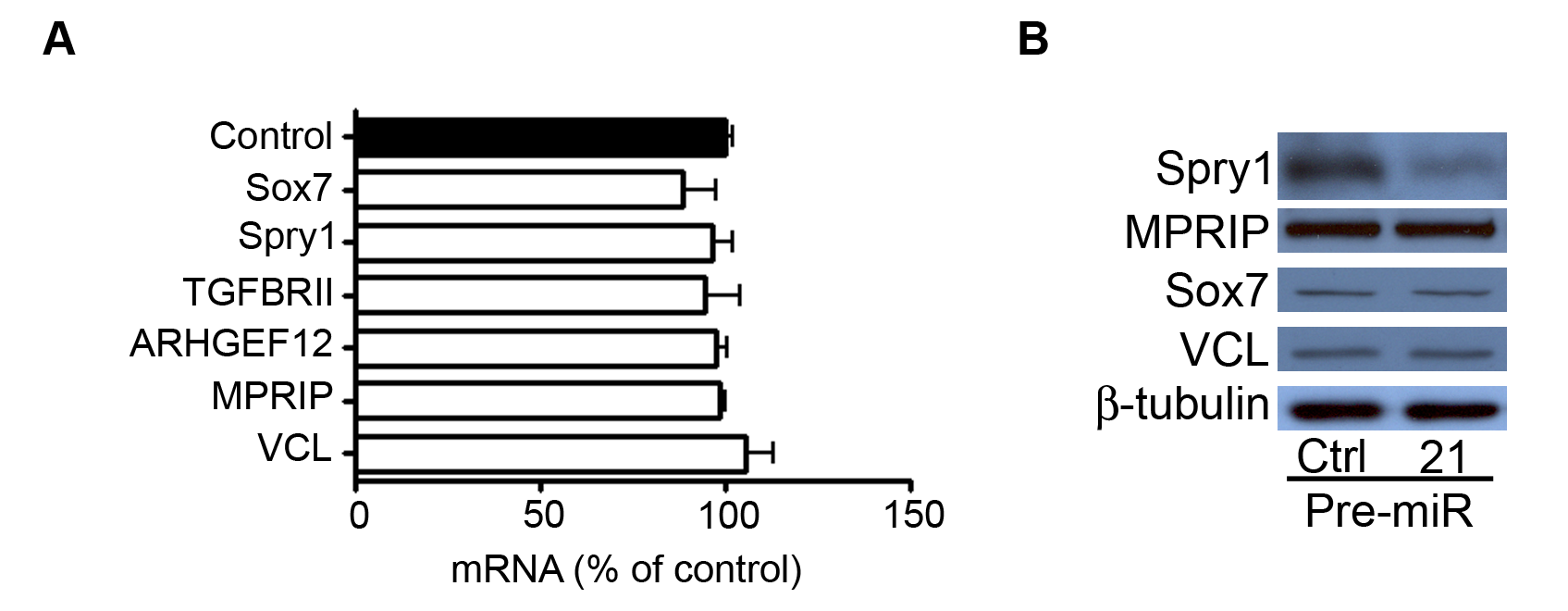

Supplement: Figure S3 — Regulation of SOX7 , TGFBRII , ARHGEF12 , MPRIP , VCL and SPRY1 expression by miR-21. A. HUVECs were transfected with a precursor of miR-21 (Pre-21) or with a precursor control (Pre-Ctrl) for 48 h. SOX7, SPRY1, TGFBRII, ARHGEF12, MPRIP and VCL expression was analyzed by qRT-PCR. The data were normalized with respect to PPIA and converted using the formula 2–ΔΔCt (relative expression). Data are means with the SD. *p<0.05 versus corresponding control; (n = 3). B. Total protein was extracted from HUVECs 48 h post transfection and MPRIP, SOX7, SPRY1 and VCL protein levels were measured by Western blotting. β-tubulin was analyzed as an internal control (representative of 3 independent experiments). (TIF) [file pone.0016979.s003.tif]

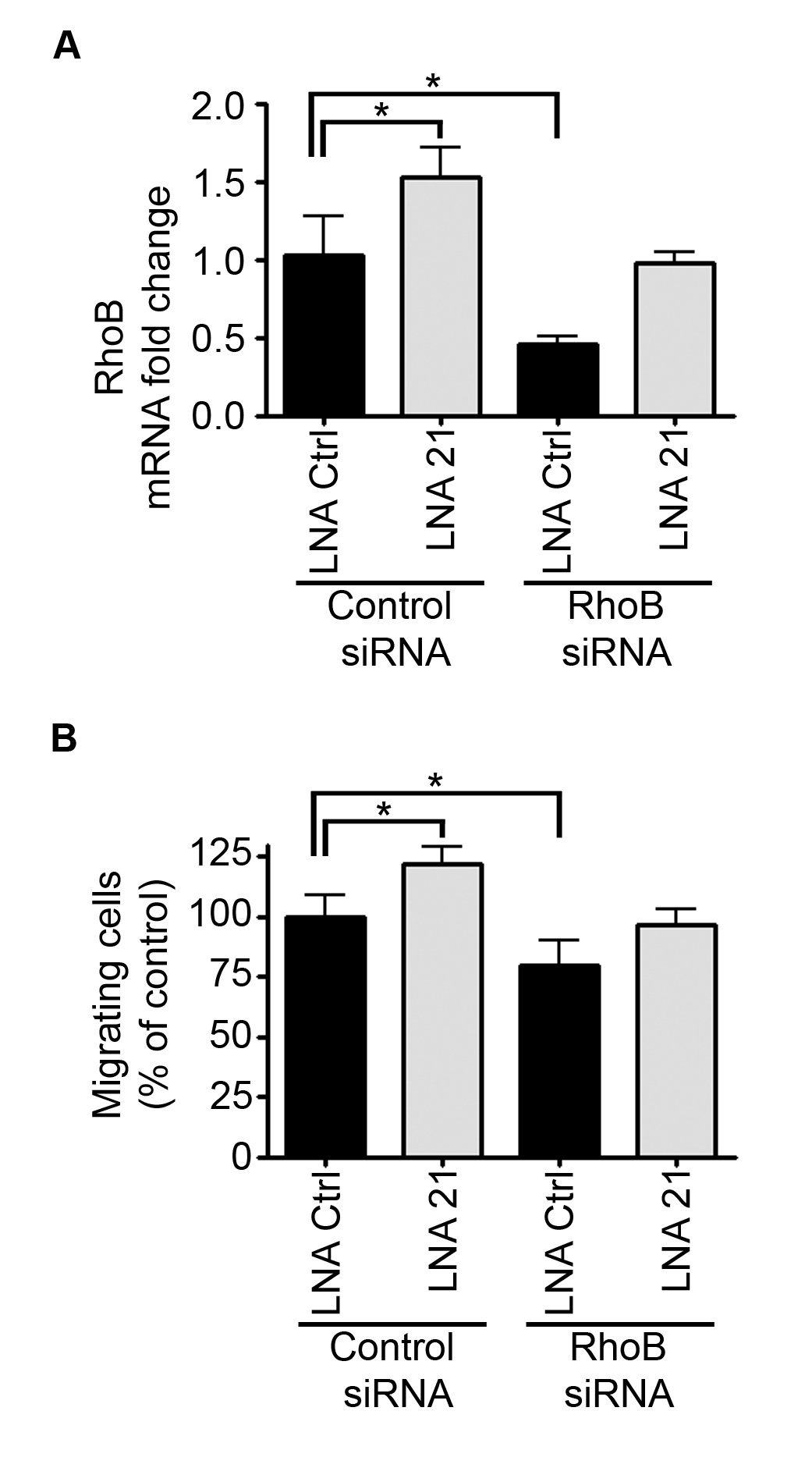

Supplement: Figure S4 — Double knockdown of miR-21 and RhoB restore RhoB expression and HUVECs migration. HUVECs were transfected with a LNA-21 or with a LNA control (LNA-Ctrl). Twenty-four hours later, HUVECs were transfected again with non-silencing siRNA (Control-siRNA) or with RhoB siRNA (RhoB siRNA). A. The RhoB mRNA level was analyzed 24 h after transfection with siRNAs by qRT-PCR. The data were normalized to GAPDH and converted using the formula 2–ΔΔCt (relative expression). B. Transfected HUVECs were assessed 48 h later for migration in a scratch-wound assay 16 h after treatment with bFGF (10 ng/ml) and VEGFa (50 ng/ml) (n = 6-12 measurements/condition; n = 3 experiments). Data are means with the SD. *p<0.05 versus corresponding control. (TIF) [file pone.0016979.s004.tif]

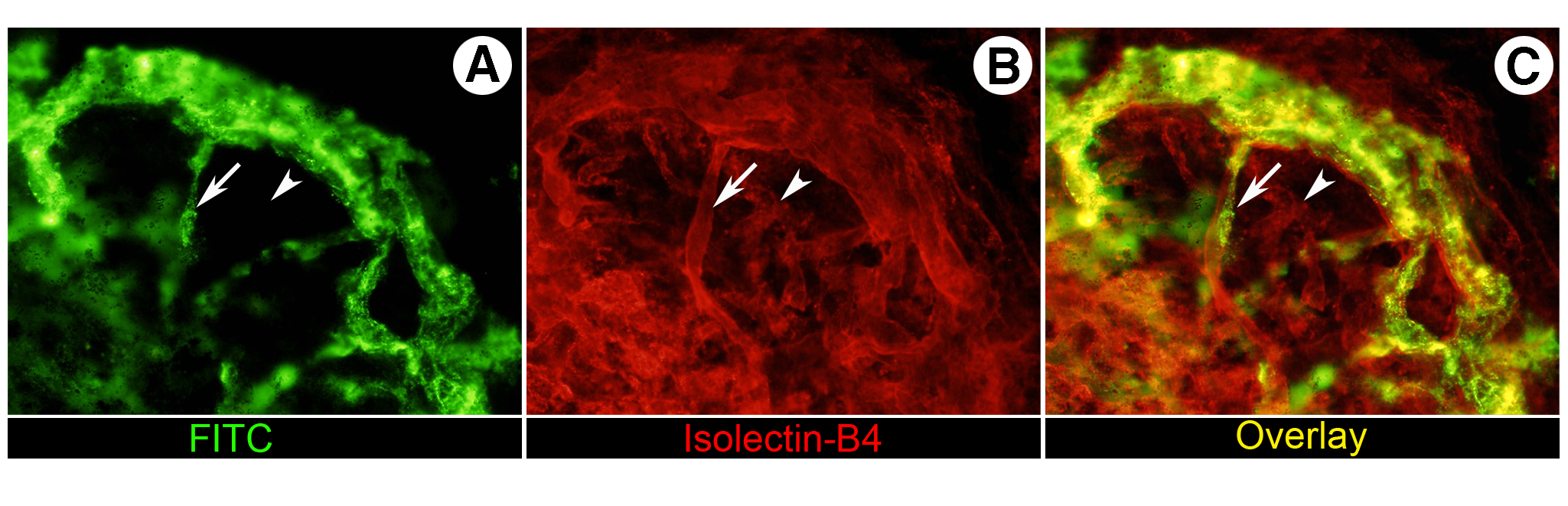

Supplement: Figure S5 — Staining of the choroidal neovascularized blood vessels. Adult C57BL/6 mice were subjected to laser-induced Bruch's membrane rupture in each eye, and then intravitreally injected with pre-miR-21 or a control molecule. Seven days after Bruch's membrane rupture at 4 locations, the mice were injected with fluorescein-labeled dextran and the eyes were removed and mounted for microscopic analysis. A. Representative picture. B. Isolectin-B4 staining of fluorescein-labeled eyes. C. Overlay of (A-B). Arrows indicate fluorescein- and isolectin-B4- labeled blood vessel. Arrowheads indicate isolectin-B4-labeled blood vessel not perfused with fluorescein. (TIF) [file pone.0016979.s005.tif]

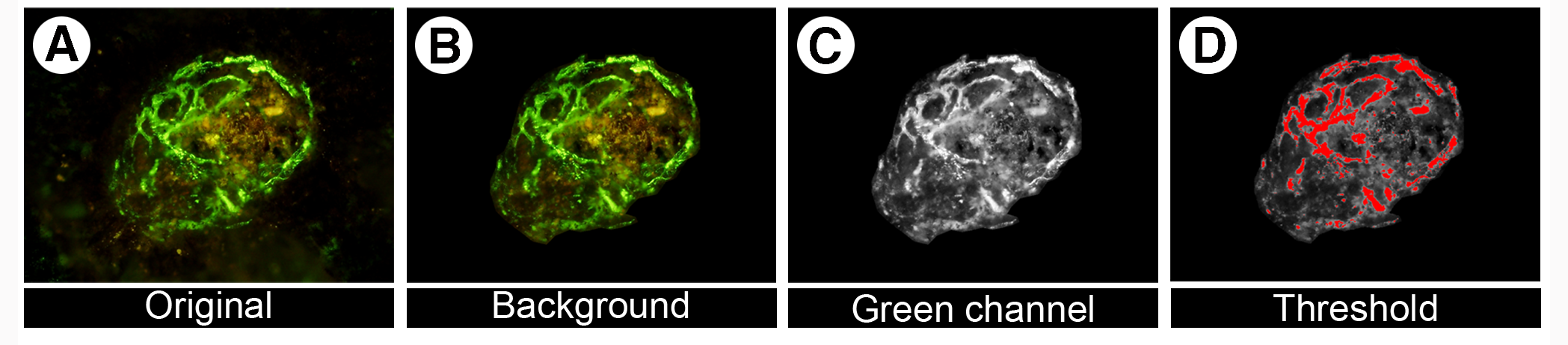

Supplement: Figure S6 — Choroidal neovascularization quantification. Adult C57BL/6 mice were subjected to laser-induced Bruch's membrane rupture in each eye, and then intravitreally injected with pre-miR-21 or a control molecule. Seven days after Bruch's membrane rupture at 4 locations, the mice were injected with fluorescein-labeled dextran and the eyes were removed and mounted for microscopic analysis. A-D. Fluorescein-labeled blood vessels quantification. A. Original Red-Green-Blue color picture. B. Selection of the blood vessels area using ImageJ erases the background from the original picture. C. Extraction of the green component. D. Quantification using ImageJ of the pixels (shown in red in the picture) after application of the threshold to mainly select blood vessels structures. (TIF) [file pone.0016979.s006.tif]
