# Supplementary material for: MicroRNA-21 Exhibits Antiangiogenic Function by Targeting RhoB Expression in Endothelial Cells
Source: PLoS One. 2011 Feb 10;6(2):e16979. doi: 10.1371/journal.pone.0016979 (PMC3037403; doi:10.1371/journal.pone.0016979)
Supplement: Table S1 — List of primers used in quantitative RT-PCR. Sequences of all primers used in qRT-PCR experiments are listed. (DOC) [file pone.0016979.s007.doc]

**Table S1 - List of primers used in quantitative RT-PCR.**

|  | Sense primers | Antisense primers |
| --- | --- | --- |
| RhoB | 5’ CCATCCGCAAGAAGCTGGA 3’ | 5’ TCTTGGCAGAGCACTCGAGGTA 3’ |
| SOX7 | 5’ CAGCGGATGCTAGAGAAGATGG 3’ | 5’ TCCGAGTCCCAGGTGGTTATTT 3’ |
| SPRY1 | 5’ GCCTTCTTTGGATAGCCGTCAG 3’ | 5’ TCATTGCTGCCTCTTATGGCC 3’ |
| MPRIP | 5’ CCACGGTGTCCGGATATGATA 3’ | 5’ GGCCTTCCTTCAGACTCTTGGA 3’ |
| ARHGEF12 | 5’ TTACTAAAATCTCGCCCGGC 3’ | 5’ TGCGACGAGTTTCTTTGGAA 3’ |
| TGFBRII | 5’ CGCACGTTCAGAAGTCGGTTA 3’ | 5’ TCTGGTTGTCACAGGTGGAAAA 3’ |
| VCL | 5’ AAGCACAGCGGTGGATTGA 3’ | 5’ GGCCCCATCATAACATTAGCC 3’ |
| PPIA | 5’ CCAACACAAATGGTTCCCAGT 3’ | 5’ CCATGGCCTCCACAATATTCA 3’ |
| GAPDH | 5’ GCATCTTCTTTTGCGTCGC 3’ | 5’ CCAAATGCGTTGACTCCGA 3’ |
